# Abnormal Development of Glutamatergic Synapses Afferent to Dopaminergic Neurons of the Pink1^−/−^ Mouse Model of Parkinson’s Disease

**DOI:** 10.3389/fncel.2016.00168

**Published:** 2016-06-23

**Authors:** Edouard Pearlstein, François J. Michel, Laurène Save, Diana C. Ferrari, Constance Hammond

**Affiliations:** ^1^UMR901, Aix-Marseille UniversitéMarseille, France; ^2^Institut de Neurobiologie de la Méditerranée (INMED), Inserm UMR 901Marseille, France

**Keywords:** pink1-deficient mouse, substantia nigra, dopaminergic neurons, development, spontaneous AMPA EPSCs, spontaneous NMDA EPSCs, patch clamp, Parkinson’s disease

## Abstract

In a preceding study, we showed that in adult pink1^−/−^ mice, a monogenic animal model of Parkinson’s disease (PD), striatal neurons display aberrant electrical activities that precede the onset of overt clinical manifestations. Here, we tested the hypothesis that the maturation of dopaminergic (DA) neurons of the pink1^−/−^ substantia nigra compacta (SNc) follows, from early stages on, a different developmental trajectory from age-matched wild type (wt) SNc DA neurons. We used immature (postnatal days P2–P10) and young adult (P30–P90) midbrain slices of pink1^−/−^ mice expressing the green fluorescent protein in tyrosine hydroxylase (TH)-positive neurons. We report that the developmental sequence of N-Methyl-D-aspartic acid (NMDA) spontaneous excitatory postsynaptic currents (sEPSCs) is altered in pink1^−/−^ SNc DA neurons, starting from shortly after birth. They lack the transient episode of high NMDA receptor-mediated neuronal activity characteristic of the immature stage of wt SNc DA neurons. The maturation of the membrane resistance of pink1^−/−^ SNc DA neurons is also altered. Collectively, these observations suggest that electrical manifestations occurring shortly after birth in SNc DA neurons might lead to dysfunction in dopamine release and constitute an early pathogenic mechanism of PD.

## Introduction

Dopaminergic (DA) neurons of the substantia nigra compacta (SNc) degenerate progressively during the course of Parkinson’s disease (PD; Kordower et al., 2013). Whether and when they dysfunction during the early stages of PD before degenerating remains largely unknown. Addressing this question requires progressive animal models of PD, like genetic models of familial forms of PD. Particularly interesting are pink1-deficient mice, which do not express the PTEN-induced kinase 1 (pink1), a ubiquitously expressed mitochondrial kinase consisting of 581 amino acids that encode for a mitochondrial targeting sequence, a transmembrane domain and a Ser/Thr kinase domain (Silvestri et al., 2005; Gandhi et al., 2006; Zhou et al., 2008; Gispert et al., 2009). Pink1 is a mitochondrial quality control factor with functions in repair, fission and autophagic elimination (Deng et al., 2008; Poole et al., 2008; Yang et al., 2008; Gehrke et al., 2015). Pink1 induces mitophagy. Upon mitochondrial damage, pink1 is stabilized on the outer mitochondrial membrane, where it phosphorylates ubiquitin and activates the ubiquitin ligase parkin. This builds ubiquitin chains on mitochondrial outer membrane proteins and leads to removal of damaged mitochondria by autophagy (Narendra et al., 2010; Koyano et al., 2014; Kazlauskaite and Muqit, 2015; Lazarou et al., 2015; Ordureau et al., 2015).

Pink1-deficient mice are a model of the PARK6 variant of PD (mutations in *PINK1*), the second most common cause of autosomal recessive familial early-onset PD (Bentivoglio et al., 2001; Valente et al., 2004; Bonifati et al., 2005; Gasser, 2009). In this model, the first conspicuous motor abnormalities are manifest at 16 months, offering a large time window to explore earlier subclinical abnormalities (Gispert et al., 2009). During this window, profound dysfunction is observed in the basal ganglia system. Though pink1^−/−^ DA SNc neurons are preserved during the murine lifespan, they progressively show mitochondrial dysfunction, impaired intracellular calcium signaling leading to functional reduction of the activation of the small K (SK) calcium-activated channels, irregular firing and a greater tendency to fire bursts of action potentials by 1–4 months (Gispert et al., 2009; Bishop et al., 2010). In addition, evoked dopamine release is reduced in the striatum, and cortico-striatal synaptic plasticity is disrupted by 3 months. Half the striatal projection neurons (medium spiny neurons, MSNs) show aberrant morphology and generate giant GABAergic currents by 3–6 months. These constitute an electrophysiological signature of dopamine depletion and PD, since they are reversed by chronic levodopa administration or subthalamic nucleus (STN) lesion used to treat PD (Kitada et al., 2007; Gautier et al., 2008; Dehorter et al., 2009, 2012; Gispert et al., 2009; Wang et al., 2011).

These observations suggest that early sub-clinical manifestations can occur, possibly leading to very early deviations in developmental processes that culminate progressively in motor disturbances. A wide range of experiments suggest that ionic currents, like the brain patterns they generate, follow developmental sequences playing specific roles in the developing brain, which when deviated lead to long-term neurological deleterious sequels (Ben-Ari, 2008). Since α-amino-3-hydroxy-5-methyl-4-isoxazolepropionic acid (AMPA) and N-methyl-D-aspartic acid (NMDA) receptor activation generates transient high-frequency activity in SNc DA neurons, alterations of their properties during development could produce long-term alterations in SNc DA neurons. Our finding that the developmental sequence of NMDA currents and membrane resistance are altered in pink1^−/−^ SNc DA neurons could explain dysfunction in dopamine release later in adulthood (Schmitz et al., 2009).

## Materials and Methods

All experiments were approved by the Institut National de la Santé et de la Recherche Médicale (INSERM) animal care and use agreement (D-13-055-19) and the European community council directive (2010/63/UE). Animals had access to food and water *ad libitum* and were housed in our institutional animal facilities under a 12 h light/dark cycle at 22–24°C.

### Mice

We used immature (P2–10) and young adult (P30–90) tyrosine hydroxylase (TH)-GFP mice as control wild type (wt) mice (Pearlstein et al., 2015) and pink1-deficient TH-GFP mice as pink1^−/−^ mice. To generate pink1^−/−^ TH-GFP mice, TH-GFP mice of the 129/Sv background were interbred with pink1^−/−^ mice of the same background. Pink1^−/−^ TH-GFP mice were subsequently identified using PCR-based genotyping. Then pink1^−/−^ TH-GFP mice were regularly crossed with pink1^−/−^ mice to generate pups. At P2–P5, TH-GFP and pink1^−/−^ TH-GFP pups were differentiated from wt pups via a UV lamp (see Pearlstein et al., 2015).

For slice preparation, drugs, cell labeling and TH immunocytochemistry, see Pearlstein et al. (2015).

#### Electrophysiology

All recordings were made in whole-cell voltage-clamp configuration. *R*_m_ and *C*_m_ were measured during a 800 ms/−10 mV step from holding potential (−60 mV). *R*_m_ was calculated using the following formula: *R*_m_ = Δ*V*(1/Δ*I*_slow_ − 1/Δ*I*_max_), where Δ*V* is the amplitude of the voltage step, Δ*I*_max_ is the difference between holding current value and the peak intensity reached by the capacitive current at the start of the hyperpolarizing pulse, and Δ*I*_slow_ is the difference in current intensity between holding current value and that of the steady-state current measured after the capacitive current. *C*_m_ was calculated as: *C*_m_ = τ_w_/*R*_m_ where τ_w_ is the weighted membrane time constant (Marcaggi et al., 2003). We measured Ih amplitude by subtracting the amplitude of the current at the end of the 800 ms hyperpolarizing step to −140 mV (*V*_H_ = −60 mV) from the amplitude of the current 15 ms after the first capacitative current. We measured spontaneous AMPA/Kainate (KA) currents in voltage-clamp mode at *V*_H_ = −60 mV in the continuous presence of Gabazine (5 μM) to block GABA_A_ receptors. We measured spontaneous NMDA currents in voltage-clamp mode at *V*_H_ = +40 mV in the continuous presence of Gabazine (5 μM) and NBQX (10 μM) to block GABA_A_ and AMPA/KA receptors, respectively. These currents were stored on a computer using Pclamp8 software (Molecular Devices) and analyzed off-line with a Mini Analysis software (Synaptosoft 6.0), to determine the inter-event intervals (IEIs), amplitude, rise time and decay time of spontaneous currents. The decay of spontaneous synaptic currents was well fitted by a single-exponential function, starting at the peak of the current to the time point when the current had decayed to 99.9% of its peak amplitude. All detected currents were then visually inspected to reject artifactual events. NMDA spontaneous excitatory postsynaptic currents (sEPSCs) occurred either as single events or in bursts. We defined a burst of NMDA sEPSCs as the occurrence of at least three superimposed NMDA sEPSCs and a bursty pattern as at least two bursts/cell/3 min. Miniature AMPA or NMDA currents, recorded in the presence of Tetrodotoxin (TTX; 1 μM), were not studied because they had an extremely low frequency at all ages tested.

#### Statistics

Results are given as mean ± standard error of the mean. The non-parametric Mann-Whitney test (Graphpad Prism 6 software, San Diego, CA, USA) was used to compare results from Pink1^−/−^ to wt SNc DA neurons (at P2–3, P4–10 and P30+). Since amplitude, IEI, rise time and decay time of sEPSCs were not normally distributed, we also calculated the median value of these parameters (± standard error of the mean of medians) for each cell (data not shown). Statistical significance was not different when comparing means or medians. We pooled the results obtained between P4 and P10 because there was no significant difference between the full set of results obtained at P4–5 and at P8–10 for AMPA- and NMDA-mediated sEPSCs (data not shown). For example, there was no significant difference in frequency (*P* = 0.3; Mann-Whitney test), and amplitude (*P* = 0.5; Mann-Whitney test) of AMPA sEPSCs. Similarly, there was no significant difference in IEI, (*P* = 0.35; Mann-Whitney test) and amplitude (*P* = 0.52; Mann-Whitney test) of single NMDA sEPSCs. We used the χ^2^ or Fisher’s exact test to compare proportions. For each test performed, the *P* value was provided and the statistical significance was set at *P* ≤ 0.05. In all figures: *P < 0.05, **P < 0.01 and ***P < 0.001.

## Results

### Intrinsic Membrane Properties of Pink1^−/−^ SNc DA Neurons did not Develop in the Same Way as those of wt

The somato-dendritic field of pink1^−/−^ SNc DA neurons did not significantly change between P4–10 (*n* = 19) and P30+ (*n* = 14), as already described for wt SNc DA neurons. The mean surface area of somas was 385 ± 40 μm^2^ at P4–10 and 227 ± 18 μm^2^ at P30+. The mean number of dendritic trunks was 4.4 ± 0.3 at P4–10 and 4.6 ± 0.3 at P30+ (*P* = 0.6), the mean number of dendritic ends was 13 ± 1 at P4–10 and 11 ± 1 at P30+ (*P* = 0.5), the mean total dendritic length was 1535 ± 179 μm at P4–10 and 1336 ± 130 at P30+ (*P* = 0.5) and the mean dendritic volume was 4.5 × 10^6^ ± 0.9 × 10^6^ μm^3^ at P4–10 and 5.4 × 10^6^ ± 1.2 × 10^6^ at P30 (*P* = 0.5). The axon originated either from the soma (*n* = 13/18 at P4–10; *n* = 3/14 at P30+) or from a primary dendritic trunk (*n* = 5/18 at P4–10; *n* = 11/14 at P30+), as already described for wt P4-P50 SNc DA neurons (Häusser et al., 1995; Gentet and Williams, 2007; Blythe et al., 2009; Pearlstein et al., 2015). Compared to age-matched somas surfaces or dendritic trees of wt SNc DA neurons (Pearlstein et al., 2015), there was no significant difference in any of the above parameters (data not shown).

**Figure 1:**
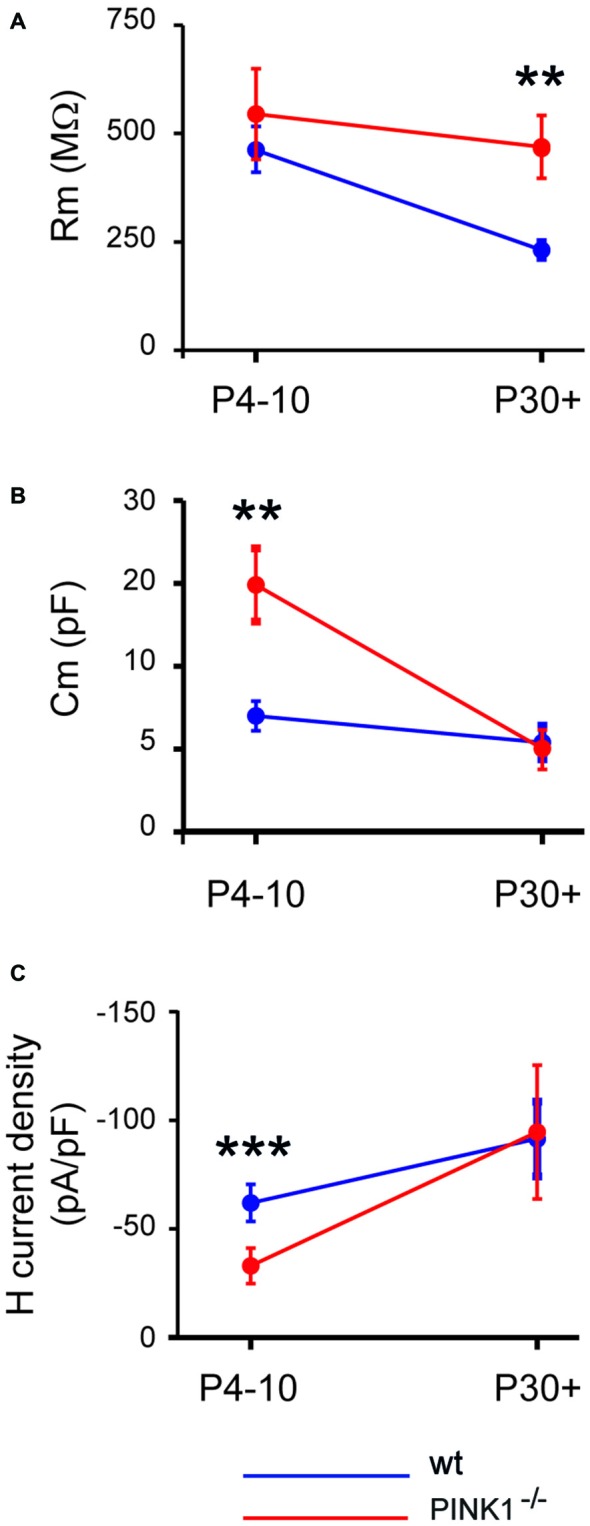
**Developmental profile of membrane resistance (A), membrane capacitance (B) and H current density (C) in pink1^−/−^ (red) and wild type (wt; blue) Substantia nigra compacta (SNc) dopaminergic (DA) neurons**.

At immature stage (P4–10), the mean membrane resistance of pink1^−/−^ SNc DA neurons (545 ± 104 MΩ, *n* = 37) was similar (*P* = 0.96) to that of wt (469 ± 54 MΩ, *n* = 27). However at young adult stage (P30+), the membrane resistance of pink1^−/−^ SNc DA neurons was still the same (462 ± 72 MΩ, *n* = 23; *P* = 0.8), while that of wt had dramatically diminished (231 ± 18 MΩ, *n* = 17) and was twice lower (*P* = 0.003; Figure 1A). Mean C_m_ of pink1^−/−^ SNc DA neurons at P4–10 was twice that of age-matched wt SNc DA neurons (15.0 ± 2.2 pF *n* = 37 vs. 7 ± 0.9 pF, *n* = 27; *P* = 0.007) while at P30+, C_m_ of pink1^−/−^ and wt were not different (5.0 ± 1.2 pF, *n* = 24 vs. 5.4 ± 1.02 pF, *n* = 17; *P* = 0.24; Figure 1B).

The H current density (*I*_h_/C_m_) was significantly smaller (*P* < 0.0001) in P4–10 Pink1^−/−^ SNc DA neurons than in wt (−32.9 ± 8.1 pA/pF, *n* = 27 vs. −61.8 ± 8.1 pA/pF). However at P30+, pink1^−/−^ and wt SNc DA neurons had comparable (*P* = 0.22) *I*_h_ current densities (−94.8 ± 30.8 pA/pF, *n* = 23 vs. −91.6 ± 17.2 pA/pF, *n* = 17; Figure 1C).

### AMPA Receptor-Mediated sEPSCs of Pink1^−/−^ SNc DA Neurons Follow the Same Postnatal Developmental Sequence as wt SNc DA Neurons

As wt SNc DA neurons (Pearlstein et al., 2015), neither immature nor young adult pink1^−/−^ SNc DA neurons generate spontaneous KA-receptor-mediated EPSCs.

**Figure 2:**
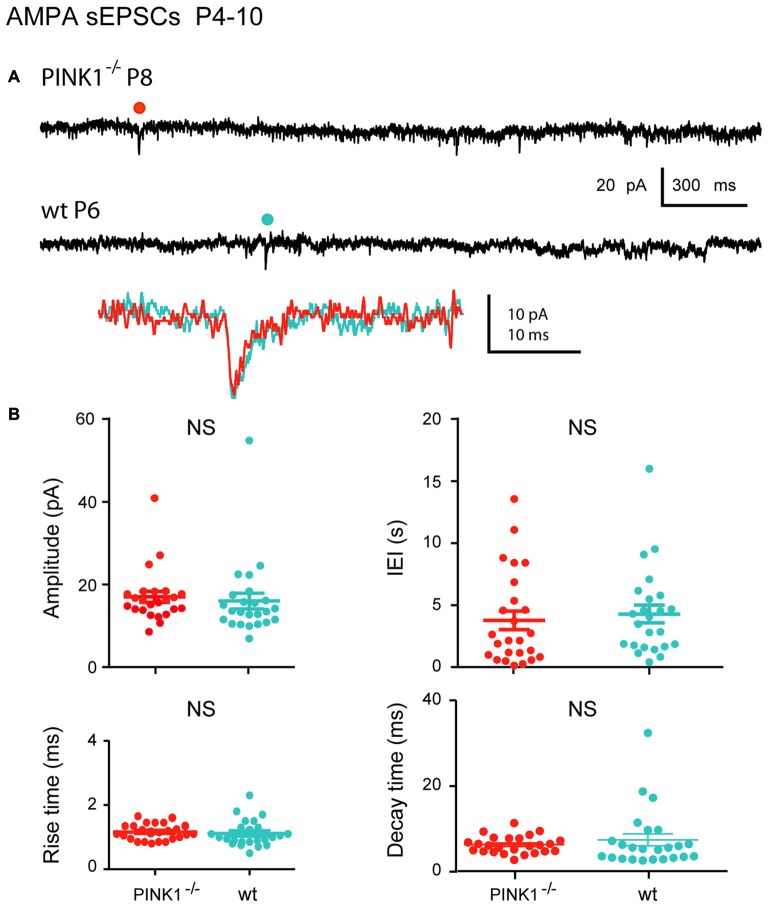
**α-amino-3-hydroxy-5-methyl-4-isoxazolepropionic acid (AMPA) spontaneous excitatory postsynaptic currents (sEPSCs) in immature (P4–10) pink1^−/−^ SNc DA neurons and comparison with age-matched wt SNc DA neurons. (A)** Voltage-clamp recordings of AMPA sEPSCs from a P8 pink1^−/−^ (red) and a P6 wt (blue) SNc DA neurons in the continuous presence of gabazine (5 μM) and APV (40 μM) at *V*_H_ = −60 mV. AMPA events indicated by a dot are enlarged and superimposed in the inset. **(B)** Quantification and statistical comparison of the amplitudes and inter-event intervals (IEIs) and of the rise and decay times of P4–10 pink1^−/−^ (*n* = 25) and wt (*n* = 24) AMPA sEPSCs, as indicated.

**Figure 3:**
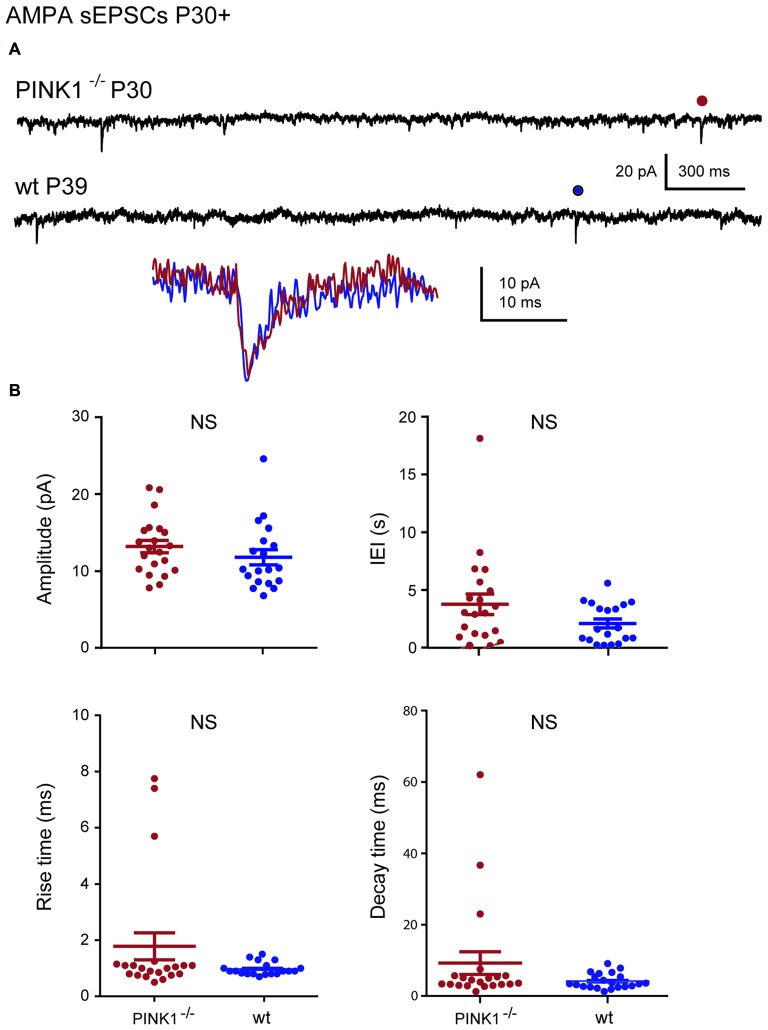
**AMPA sEPSCs in young adult (P30+) pink1^−/−^ SNc DA neurons and comparison with age-matched wt SNc DA neurons. (A)** Voltage-clamp recordings of AMPA sEPSCs from a P30 pink1^−/−^ (red) and a P39 wt (blue) SNc DA neuron in the continuous presence of gabazine (5 μM) and APV (40 μM) at *V*_H_ = −60 mV. AMPA events indicated by a dot are enlarged and superimposed in the inset. **(B)** Quantification and statistical comparison of the amplitudes and IEIs and of the rise and decay times of P30+ pink1^−/−^ (*n* = 21) and wt (*n* = 19) AMPA sEPSCs, as indicated.

At immature stage (P4–10), AMPA receptor-mediated sEPSCs (AMPA sEPSCs) of pink1^−/−^ SNc DA neurons had similar mean amplitudes and lengths of IEIs (17.2 ± 1.3 pA; 3.9 ± 0.7 s; *n* = 25) to those of wt (16.0 ± 1.9 pA; 4.4 ± 0.7 s; *n* = 24; *P* = 0.1; *P* = 0.32;). Their mean rise (1.26 ± 0.06 ms in pink1^−/−^; 1.17 ± 0.08 ms in wt) and decay (8.0 ± 0.5 ms in pink1^−/−^; 9.2 ± 1.6 ms in wt) times were also similar (*P* = 0.23; *P* = 0.36; Figures 2A,B). At young adult stage (P30+), mean amplitudes and IEIs of P30+ AMPA sEPSCs were similar in pink1^−/−^ (13.1 ± 0.8 pA; 3.8 ± 0.9 s; *n* = 21) and wt (11.8 ± 1.0; 2.1 ± 0.4 s; *n* = 19) SNc DA neurons (*P* = 0.2; *P* = 0.5). Mean rise (1.8 ± 0.5 ms in pink1^−/−^; 1.11 ± 0.09 ms in wt) and decay (5.0 ± 0.5 ms in pink1^−/−^; 5.8 ± 1.0 ms in wt) times (*P* = 0.9; *P* = 0.9) were also similar (Figures 3A,B). We have previously shown that wt immature AMPA sEPSCs had larger amplitudes and longer IEIs than young adult ones (Pearlstein et al., 2015). AMPA sEPSCs of immature pink1^−/−^ SNc DA neurons also had a significantly larger mean amplitude than did young adult neurons (*P* = 0.005; see Figure 7A). In contrast, the mean length of IEIs of AMPA sEPSCs of pink1^−/−^ SNc DA neurons did not differ significantly between P4–10 and P30+ (*P* = 0.45; Figures 2B, 3B, see Figure 7B).

### The wt Developmental Sequence of NMDAR-Mediated Spontaneous Currents is Abolished in Pink1^−/−^ SNc DA Neurons

In this set of experiments, we first observed at P4–10 a drastic change in the developmental sequence of NMDA sEPSCs in pink1^−/−^ compared to wt SNc DA neurons. To determine whether this developmental sequence had shifted towards younger ages, we recorded NMDA sEPSCs at P2–3 in both wt and mutated mice.

**Figure 4:**
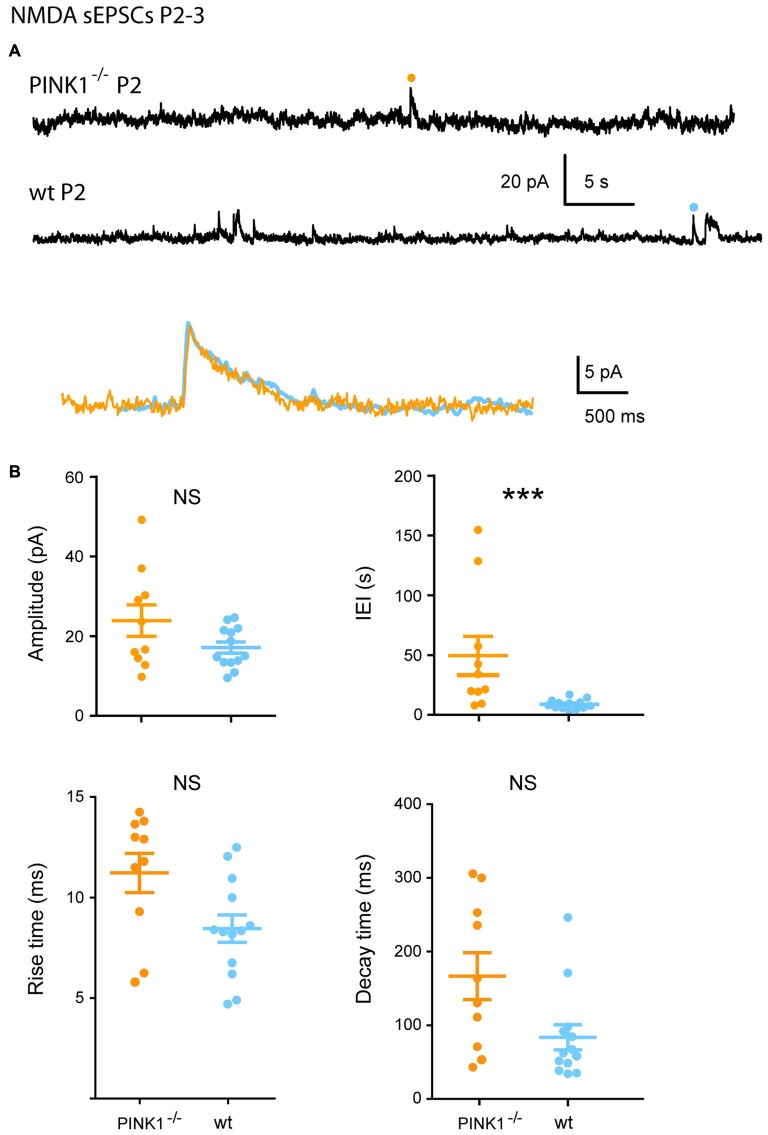
**Single N-methyl-D-aspartic acid (NMDA) sEPSCs P2–3 pink1^−/−^ SNc DA neurons and comparison with age-matched wt SNc DA neurons. (A)** Voltage-clamp recordings of NMDA sEPSCs from a P2 pink1^−/−^ (orange) and a P2 wt (turquoise blue) SNc DA neuron in the continuous presence of gabazine (5 μM) and NBQX (10 μM) at *V*_H_ = +40 mV. The NMDA events indicated by a dot are enlarged and superimposed in the inset. **(B)** Quantification and statistical comparison of the amplitudes and IEIs and of the rise and decay times of P2–3 pink1^−/−^ (*n* = 10) and wt (*n* = 13) NMDA sEPSCs, as indicated.

At P2–3, 59% of pink1^−/−^ (10/17) vs. 100% (13/13) of wt SNc DA neurons generated NMDA sEPSCs. Single P2–3 pink1^−/−^ NMDA sEPSCs had similar mean amplitudes (23.3 ± 4.0 pA; *n* = 10; *P* = 0.23) but much longer mean IEIs (48.6 ± 16.0 s; *P* = 0.0006) than age-matched wt DA neurons (16.6 ± 1.4 pA; 8.3 ± 1.1 s; *n* = 13). As they were very immature signals, these NMDA sEPSCs had very long mean rise (pink1^−/−^: 12.3 ± 0.9 ms; wt: 11.0 ± 0.6 ms; *P* = 0.1) and decay (pink1^−/−^: 224.6 ± 43.4 ms; wt: 187.1 ± 30.2 ms; *P* = 0.5) times (Figures 4A,B).

**Figure 5:**
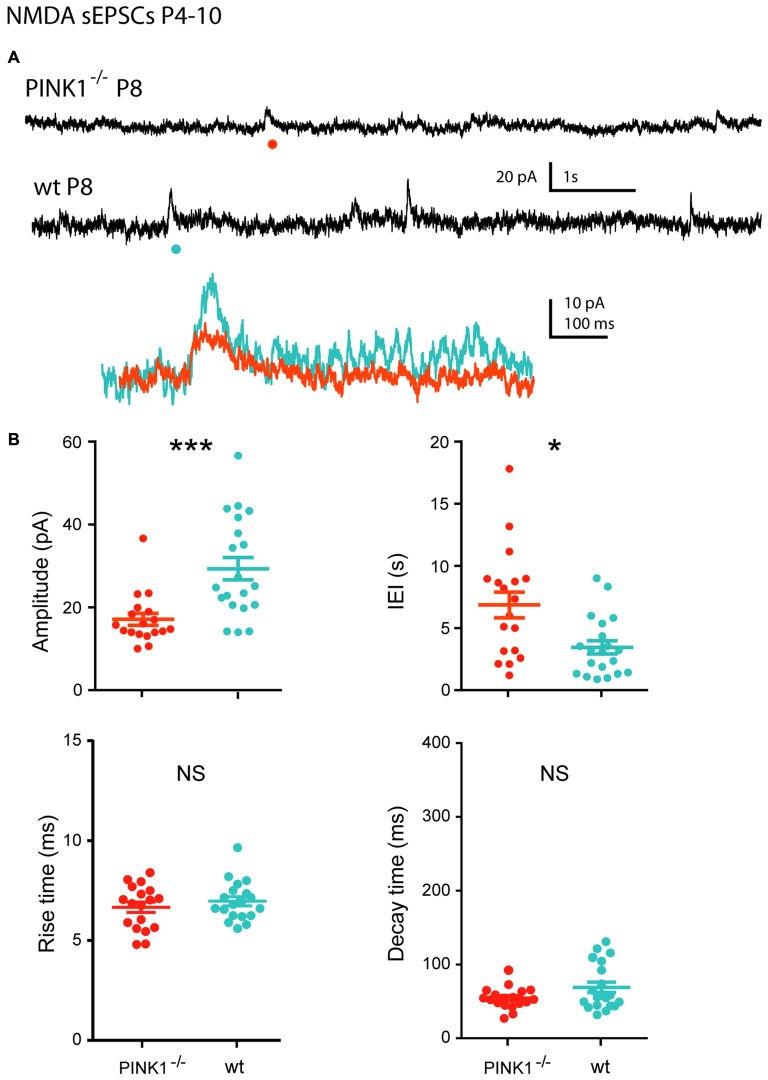
**Single NMDA sEPSCs in immature (P4–10) pink1^−/−^ SNc DA neurons and comparison with age-matched wt SNc DA neurons. (A)** Voltage-clamp recordings of NMDA sEPSCs from a P8 pink1^−/−^ (red) and a P8 wt (blue) SNc DA neuron in the continuous presence of gabazine (5 μM) and NBQX (10 μM) at *V*_H_ = +40 mV. The NMDA events indicated by a dot are enlarged and superimposed in the inset. **(B)** Quantification and statistical comparison of the amplitudes and IEIs and of the rise and decay times of P4–10 pink1^−/−^ (*n* = 18) and wt (*n* = 20) NMDA sEPSCs, as indicated.

At P4–10, single NMDA receptor-mediated sEPSCs (NMDA sEPSCs) of P4–10 pink1^−/−^ SNc DA neurons had significantly smaller amplitudes and longer IEIs (17.1 ± 1.4 pA; 6.8 ± 1.0 s; *n* = 18) than wt ones (29.3 ± 2.7 pA; 3.6 ± 0.6 s; *n* = 20; *P* = 0.0002; *P* = 0.012). In contrast, their mean rise (7.0 ± 0.2 ms in pink1^−/−^; 7.3 ± 0.2 ms in wt) and decay (81.4 ± 5.5 ms in pink1^−/−^; 101.5 ± 3.8 ms in wt) times were similar (*P* = 0.8; *P* = 0.1; Figures 5A,B).

**Figure 6:**
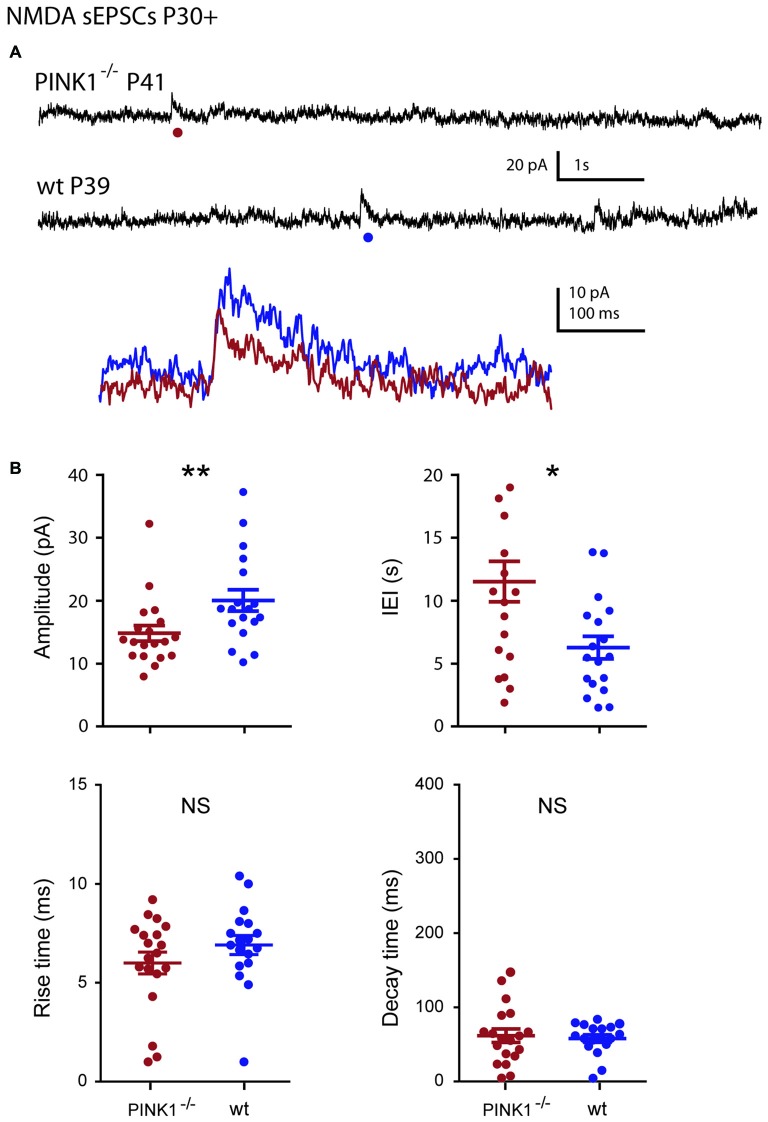
**Single NMDA sEPSCs in young adult (P30+) pink1^−/−^ SNc DA neurons and comparison with age-matched wt SNc DA neurons. (A)** Voltage-clamp recordings of NMDA sEPSCs from a P41 pink1^−/−^ (red) and a P39 wt (blue) SNc DA neuron in the continuous presence of gabazine (5 μM) and NBQX (10 μM) at *V*_H_ = +40 mV. The NMDA events indicated by a dot are enlarged and superimposed in the inset. **(B)** Quantification and statistical comparison of the amplitudes and IEIs and of the rise and decay times of P30+ pink1^−/−^ (*n* = 19) and wt (*n* = 18) NMDA sEPSCs, as indicated.

**Figure 7:**
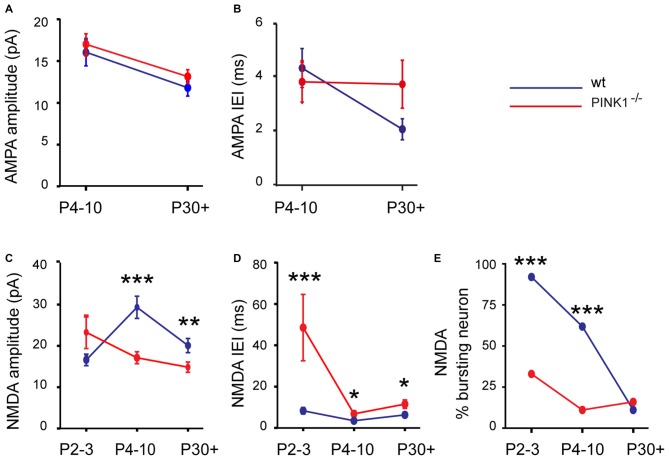
**Developmental profile in pink1^−/−^ (red) and wt (blue) SNc DA neurons of the amplitude (A) and IEIs (B) of AMPA sEPSCs; of the amplitude (C) and IEIs (D) of NMDA sEPSCs; of the percentage of neurons with bursts of NMDA sEPSCs (E)**.

At young adult stage (P30+), the situation was comparable. Amplitude and IEIs of P30+ NMDA sEPSCs were significantly smaller and longer in pink1^−/−^ (14.8 ± 1.2 pA; 11.5 ± 1.6 s; *n* = 19) than in wt (20.0 ± 1.7 pA; 6.3 ± 0.9 s; *n* = 18; *P* = 0.005; *P* = 0.014) DA SNc neurons. But their mean rise (6.8 ± 0.5 ms in pink1^−/−^; 7.6 ± 0.4 ms in wt) and decay (86.1 ± 7.3 ms in pink1^−/−^; 75.5 ± 4.5 ms in wt) times were similar (*P* = 0.3; *P* = 0.8; Figures 6A,B). We previously showed that wt NMDA sEPSCs had larger amplitudes and shorter IEIs at P4–10 than at P30+ (Pearlstein et al., 2015). The situation was strikingly different for the amplitude of pink1^−/−^ NMDA sEPSCs neurons, being similar in the two age groups (*P* = 0.9; see Figure 7C). Only the mean lengths of IEIs differed significantly between the two age groups (IEI were shorter at P4–10 than at P30+; *P* = 0.033; see Figure 7D).

Immature pink1^−/−^ NMDA sEPSCs rarely occurred in bursts (involving 3–5 events), in contrast to the age-matched wt group (Pearlstein et al., 2015). There were far fewer (*P* < 0.001, χ^2^ test) pink1^−/−^ SNc DA neurons showing a bursty pattern of NMDA sEPSCs (see “Materials and Methods” Section) at P2–3 (30%, *n* = 3/10, 35 bursts in 3 neurons) than age-matched wt ones (92%, *n* = 12/13, 86 bursts in 12 neurons). Bursts of NMDA sEPSCs were present in only 11% (2/18) of P4–10 pink1^−/−^ SNc DA neurons (5 bursts in 2 neurons) compared to 60% (12/20) in wt SNc DA neurons (146 bursts in 12 neurons; *P* < 0.001, χ^2^ test). In young adults (P30+), the percentage of bursting DA neurons was comparable (*P* = 1, Fisher’s exact test) in pink1^−/−^ (16%; 3/19 neurons, 26 bursts in 3 neurons) and wt (11%; 2/18 neurons; 26 bursts in 3 neurons) SNc (see Figure 7E).

#### Lack of GluN2C/D-Containing NMDARs in Immature Pink1^−/−^ SNc Neurons

In contrast to wt, Pink1^−/−^ NMDA sEPSCs did not show different pharmacological sensitivity with age (Pearlstein et al., 2015). DQP 1105 (10 μM), a preferential antagonist of GluN2D-containing NMDA receptors (previously termed NR2D in rodents and corresponding to GRIN2D in humans), had no effect on the amplitude (21.8 ± 5.1 pA before and 24.4 ± 5.0 pA during DQP, *n* = 5; *P* = 0.06) and frequency (0.25 ± 0.07 Hz before and 0.20 ± 0.02 Hz during DQP, *n* = 5; *P* = 0.3) of P4–10 single pink1^−/−^ NMDA sEPSCs. DQP too had no effect on the amplitude and frequency of P30+ single pink1^−/−^ NMDA sEPSCs (14.7 ± 1.3 pA before and 13.6 ± 1.4 pA during DQP, *n* = 6, *P* = 0.6; 0.21 ± 0.10 Hz before and 0.31 ± 0.14 Hz during DQP, *n* = 6, *P* = 0.5; data not shown). Since very few pink1^−/−^ SNc neurons had a bursty pattern at P4–10 or P30+, we did not test DQP on burst characteristics. These results show the lack of effect of DQP 1105 (10 μM) in immature pink1^−/−^ SNc DA neurons. This differs radically from findings for wt P4–10 SNc DA neurons, where DQP reduced both the frequency of single NMDA sEPSCs and the occurrence of bursts of P4–10 NMDA sEPSCs (Pearlstein et al., 2015). Ro 25-6981 (1 μM) abolished all single and bursting NMDA sEPSCs in P4–10 and P30+ pink1^−/−^ SNc neurons (data not shown). The above results also suggest that all spontaneously activated NMDA receptors, from P4 to P90 pink1^−/−^ SNc DA neurons, contained the GluN2B subunit.

## Discussion

The main result shown here is that pink1^−/−^ SNc DA neurons lack the characteristic wt developmental sequence of NMDA sEPSCs identified between P2 and P10, which consists of large amplitude and high frequency events associated to a characteristic bursting pattern. The absence of this high NMDA activity at immature stage drastically changes the developmental profile of NMDA sEPSCs in pink1^−/−^ SNc DA neurons. This may have consequences on calcium influxes into developing dendrites and somas. In contrast, there is a similar developmental profile of AMPA sEPSCs, i.e., immature AMPA sEPSCs with larger amplitudes and longer IEIs than young adult ones, in pink1^−/−^ and wt (Pearlstein et al., 2015) SNc DA neurons. This abnormal skip in the NMDA developmental sequence recorded *in vitro* in coronal slices most probably concerns glutamatergic synapses between midbrain glutamatergic neurons located in the Ventral tegmental area (VTA) and/or in the SNc and SNc DA neurons.

Maturation of the morphological properties of wt SNc DA neurons is achieved during the very early prenatal period in mice (Ferrari et al., 2012), and rats (Tepper et al., 1994; Park et al., 2000). We found comparable morphological maturation for pink1^−/−^ SNc DA neurons, with their dendritic tree already mature at P4–10. In contrast, intrinsic membrane properties developed differently. The membrane resistance of pink1^−/−^ SNc DA neurons did not diminish between P4–10 and P30+ in the manner observed for wt neurons (Pearlstein et al., 2015), suggesting the maintenance of immature features in pink1^−/−^ SNc DA neurons. This paves the way for alterations in both intrinsic and evoked activities in pink1^−/−^ SNc DA neurons, as high input resistance facilitates the generation of oscillations.

To date, the nature of the intrinsic current underlying these changes has not been determined. Cationic H channels do not appear to be involved: the amplitude of *I*_h_ at P30+ is similar. Further experiments are required to shed light on these differences, which are clearly not the consequence of morphological features, since dendritic arborizations and soma surfaces were similar in wt and pink1^−/−^ SNc DA neurons.

The absence of large and frequent NMDA events with a bursty pattern at immature stage (P2 to P10) was the striking difference between pink1^−/−^ and wt SNc DA neurons. Even later, at young adult stage (P30–P90), synaptic NMDA activity remained limited, with smaller and less frequent NMDA sEPSCs. The absence of bursts of NMDA sEPSCs and the lack of effect of DQP 1105, a preferential antagonist of NR2D-containing NMDA receptors, confirm that the glutamatergic synapses afferent to pink1^−/−^ SNc DA neurons studied here in coronal slices are different from the wt ones. In particular the presence of bursts in control SNc DA neurons was correlated to the presence of slowly-decaying NMDA events. This strongly suggests that the dynamic remodeling of NMDA receptor subunit composition described in midbrain regions (Monyer et al., 1994; Dunah et al., 1996; Wenzel et al., 1996; Laurie et al., 1997; Liu and Wong-Riley, 2010) during postnatal development, with GluN2D subunits no longer present in young adult wt mice, is altered in pink1^−/−^ SNc DA neurons, at least for the glutamatergic synapses studied here (see paragraph below). We propose that from P2 to P90, the NMDA sEPSCs of pink1^−/−^ SNc DA neurons that we recorded in the present study result from the activation of diheteromeric GluN1/GluN2B receptors (Dunah et al., 1998; Jones and Gibb, 2005; Brothwell et al., 2008; Suarez et al., 2010; Huang and Gibb, 2014), since all NMDA sEPSCs were antagonized by Ro 25-6981 but not by DQP 1105.

The most abundant afferent glutamatergic inputs to SNc DA neurons come from the subthalamic and pedunculopontine nuclei (Hammond et al., 1978, 1983; Kita and Kitai, 1987; Smith and Grace, 1992; Iribe et al., 1999; Ammari et al., 2009). However, given that in the presence of TTX, ongoing NMDA receptor-mediated sEPSCs were absent, AMPA and NMDA spontaneous events were generated by afferent action potential firing, implying that somas of afferent glutamatergic inputs were present in the slice. In coronal slices, STN or pedunculopontine somas are absent, which makes the VTA (A10) and SNc (A9) the most likely candidate glutamatergic neuronal sources (Kawano et al., 2006; Yamaguchi et al., 2007, 2011, 2013; Stuber et al., 2010; Tecuapetla et al., 2010; Zhang et al., 2015). Interestingly, several studies linked polymorphisms of the *GRIN2B* gene (GluN2B in rodents) to the increased risk of impulse control behavior in PD patients under DA treatment (Lee et al., 2009; Zainal Abidin et al., 2015).

Summing up, our previous studies in pink1^−/−^ mice showed an excess of cortical synchronization at immature stage (P15–20), followed by giant GABAergic currents in striatal MSNs (3–6 month-old) that are reversed either by STN high frequency stimulation (Carron et al., 2014), or chronic STN lesion or levodopa treatment (Dehorter et al., 2012). These features are therefore relevant to PD and might provide biological markers of the early stages of the disease, which in this model is known to begin at 16 months (Gispert et al., 2009). This would correspond to very early insults in human brain development since postnatal days 4–10 in mice correspond in humans to the last trimester of gestation and P20 to delivery.

The underlying “neuroarcheology” concept (Ben-Ari, 2008) has now been confirmed in several developmental disorders in which genetic mutations like early environmental insults have been shown to impact the development of brain networks. We propose that the pink1 mutation deviates the developmental sequence of NMDA currents at a very early stage, leading to altered formation of functional neuronal ensembles that could later culminate in clinical manifestations. Preclinical nigrostriatal dysfunction has been previously identified in cotwins with idiopathic PD and in asymptomatic carriers of a single mutant PARK6 allele. Some of these subjects developed parkinsonian signs a few years later (Piccini et al., 1997, 1999; Khan et al., 2002). Presynaptic DA dysfunction may be a central pathogenic precursor of PD, before leading to frank loss of nigral DA neurons (Shen, 2010).

## Author Contributions

EP, FJM, LS and DF performed the experiments. EP and CH analyzed the data. CH designed the study and wrote the article.

## Conflict of Interest Statement

The authors declare that the research was conducted in the absence of any commercial or financial relationships that could be construed as a potential conflict of interest.

## References

[B1] Ammari R. Lopez C. Fiorentino H. Gonon F. Hammond C. (2009). A mouse juvenile or adult slice with preserved functional nigro-striatal dopaminergic neurons. Neuroscience 159, 3–6. 10.1016/j.neuroscience.2008.10.051 19032976

[B2] Ben-Ari Y. (2008). Neuro-archaeology: pre-symptomatic architecture and signature of neurological disorders. Trends Neurosci. 31, 626–636. 10.1016/j.tins.2008.09.002 18951639

[B3] Bentivoglio A. R. Cortelli P. Valente E. M. Ialongo T. Ferraris A. Elia A.. (2001). Phenotypic characterisation of autosomal recessive PARK6-linked parkinsonism in three unrelated Italian families. Mov. Disord. 16, 999–1006. 10.1002/mds.10034 11748730

[B4] Bishop M. W. Chakraborty S. Matthews G. A. Dougalis A. Wood N. W. Festenstein R.. (2010). Hyperexcitable substantia nigra dopamine neurons in P. J.Neurophysiol. 104, 3009–3020. 10.1152/jn.00466.2010 20926611 PMC3007632

[B5] Blythe S. N. Wokosin D. Atherton J. F. Bevan M. D. (2009). Cellular mechanisms underlying burst firing in substantia nigra dopamine neurons. J. Neurosci. 29, 15531–15541. 10.1523/JNEUROSCI.2961-09.2009 20007477 PMC2834564

[B6] Bonifati V. Rohé C. F. Breedveld G. J. Fabrizio E. De Mari M. Tassorelli C.. (2005). Early-onset parkinsonism associated with PINK1 mutations: frequency, genotypes and phenotypes. Neurology 65, 87–95. 10.1212/01.wnl.0000167546.39375.82 16009891

[B7] Brothwell S. L. Barber J. L. Monaghan D. T. Jane D. E. Gibb A. J. Jones S. (2008). NR2B- and NR2D-containing synaptic NMDA receptors in developing rat substantia nigra pars compacta dopaminergic neurones. J. Physiol. 586, 739–750. 10.1113/jphysiol.2007.144618 18033813 PMC2375608

[B8] Carron R. Filipchuk A. Nardou R. Singh A. Michel F. J. Humphries M. D.. (2014). Early hypersynchrony in juvenile PINK1^−/−^ motor cortex is rescued by antidromic stimulation. Front. Syst. Neurosci. 8:95. 10.3389/fnsys.2014.00095 24904316 PMC4033197

[B9] Dehorter N. Guigoni C. Lopez C. Hirsch J. Eusebio A. Ben-Ari Y.. (2009). Dopamine-deprived striatal GABAergic interneurons burst and generate repetitive gigantic IPSCs in medium spiny neurons. J. Neurosci. 29, 7776–7787. 10.1523/JNEUROSCI.1527-09.2009 19535589 PMC6665619

[B10] Dehorter N. Lozovaya N. Mdzomba B. J. Michel F. J. Lopez C. Tsintsadze V.. (2012). Subthalamic lesion or levodopa treatment rescues giant GABAergic currents of PINK1-deficient striatum. J. Neurosci. 32, 18047–18053. 10.1523/JNEUROSCI.2474-12.2012 23238720 PMC6621724

[B11] Deng H. Dodson M. W. Huang H. Guo M. (2008). The Parkinson’s disease genes pink1 and parkin promote mitochondrial fission and/or inhibit fusion in *Drosophila*. Proc. Natl. Acad. Sci. U S A 105, 14503–14508. 10.1073/pnas.0803998105 18799731 PMC2567186

[B12] Dunah A. W. Luo J. Wang Y. H. Yasuda R. P. Wolfe B. B. (1998). Subunit composition of N-methyl-D-aspartate receptors in the central nervous system that contain the NR2D subunit. Mol. Pharmacol. 53, 429–437. 9495808 10.1124/mol.53.3.429

[B13] Dunah A. W. Yasuda R. P. Wang Y. H. Luo J. Davila-Garcia M. Gbadegesin M.. (1996). Regional and ontogenic expression of the NMDA receptor subunit NR2D protein in rat brain using a subunit-specific antibody. J. Neurochem. 67, 2335–2345. 10.1046/j.1471-4159.1996.67062335.x 8931465

[B14] Ferrari D. C. Mdzomba B. J. Dehorter N. Lopez C. Michel F. J. Libersat F.. (2012). Midbrain dopaminergic neurons generate calcium and sodium currents and release dopamine in the striatum of pups. Front. Cell. Neurosci. 6:7. 10.3389/fncel.2012.00007 22408606 PMC3297358

[B15] Gandhi S. Muqit M. M. Stanyer L. Healy D. G. Abou-Sleiman P. M. Hargreaves I.. (2006). PINK1 protein in normal human brain and Parkinson’s disease. Brain 129, 1720–1731. 10.1093/brain/awl114 16702191

[B16] Gasser T. (2009). Molecular pathogenesis of Parkinson disease: insights from genetic studies. Expert Rev. Mol. Med. 11:e22. 10.1017/s1462399409001148 19631006

[B17] Gautier C. A. Kitada T. Shen J. (2008). Loss of PINK1 causes mitochondrial functional defects and increased sensitivity to oxidative stress. Proc. Natl. Acad. Sci. U S A 105, 11364–11369. 10.1073/pnas.0802076105 18687901 PMC2516271

[B18] Gehrke S. Wu Z. Klinkenberg M. Sun Y. Auburger G. Guo S.. (2015). PINK1 and Parkin control localized translation of respiratory chain component mRNAs on mitochondria outer membrane. Cell Metab. 21, 95–108. 10.1016/j.cmet.2014.12.007 25565208 PMC4455944

[B19] Gentet L. J. Williams S. R. (2007). Dopamine gates action potential backpropagation in midbrain dopaminergic neurons. J. Neurosci. 27, 1892–1901. 10.1523/JNEUROSCI.5234-06.2007 17314285 PMC6673536

[B20] Gispert S. Ricciardi F. Kurz A. Azizov M. Hoepken H. H. Becker D.. (2009). Parkinson phenotype in aged PINK1-deficient mice is accompanied by progressive mitochondrial dysfunction in absence of neurodegeneration. PLoS One 4:e5777. 10.1371/journal.pone.0005777 19492057 PMC2686165

[B21] Hammond C. Deniau J. M. Rizk A. Féger J. (1978). Electrophysiological demonstration of an excitatory subthalamonigral pathway in the rat. Brain Res. 151, 235–244. 10.1016/0006-8993(78)90881-8 209862

[B22] Hammond C. Rouzaire-Dubois B. Féger J. Jackson A. Crossman A. R. (1983). Anatomical and electrophysiological studies on the reciprocal projections between the subthalamic nucleus and nucleus tegmenti pedunculopontinus in the rat. Neuroscience 9, 41–52. 10.1016/0306-4522(83)90045-3 6308507

[B23] Häusser M. Stuart G. Racca C. Sakmann B. (1995). Axonal initiation and active dendritic propagation of action potentials in substantia nigra neurons. Neuron 15, 637–647. 10.1016/0896-6273(95)90152-3 7546743

[B24] Huang Z. Gibb A. J. (2014). Mg2+ block properties of triheteromeric GluN1-GluN2B-GluN2D NMDA receptors on neonatal rat substantia nigra pars compacta dopaminergic neurones. J. Physiol. 592, 2059–2078. 10.1113/jphysiol.2013.267864 24614743 PMC4027860

[B25] Iribe Y. Moore K. Pang K. C. Tepper J. M. (1999). Subthalamic stimulation-induced synaptic responses in substantia nigra pars compacta dopaminergic neurons *in vitro*. J. Neurophysiol. 82, 925–933. 10444687 10.1152/jn.1999.82.2.925

[B26] Jones S. Gibb A. J. (2005). Functional NR2B- and NR2D-containing NMDA receptor channels in rat substantia nigra dopaminergic neurons. J. Physiol. 569, 209–221. 10.1113/jphysiol.2005.095554 16141268 PMC1464203

[B27] Kawano M. Kawasaki A. Sakata-Haga H. Fukui Y. Kawano H. Nogami H.. (2006). Particular subpopulations of midbrain and hypothalamic dopamine neurons express vesicular glutamate transporter 2 in the rat brain. J. Comp. Neurol. 498, 581–592. 10.1002/cne.21054 16917821

[B28] Kazlauskaite A. Muqit M. M. (2015). PINK1 and Parkin - mitochondrial interplay between phosphorylation and ubiquitylation in Parkinson’s disease. FEBS J. 282, 215–223. 10.1111/febs.13127 25345844 PMC4368378

[B29] Khan N. L. Valente E. M. Bentivoglio A. R. Wood N. W. Albanese A. Brooks D. J.. (2002). Clinical and subclinical dopaminergic dysfunction in PARK6-linked parkinsonism: an 18F-dopa PET study. Ann. Neurol. 52, 849–853. 10.1002/ana.10417 12447943

[B30] Kita H. Kitai S. T. (1987). Efferent projections of the subthalamic nucleus in the rat: light and electron microscopic analysis with the PHA-L method. J. Comp. Neurol. 260, 435–452. 10.1002/cne.902600309 2439552

[B31] Kitada T. Pisani A. Porter D. R. Yamaguchi H. Tscherter A. Martella G.. (2007). Impaired dopamine release and synaptic plasticity in the striatum of PINK1-deficient mice. Proc. Natl. Acad. Sci. U S A 104, 11441–11446. 10.1073/pnas.0702717104 17563363 PMC1890561

[B32] Kordower J. H. Olanow C. W. Dodiya H. B. Chu Y. Beach T. G. Adler C. H.. (2013). Disease duration and the integrity of the nigrostriatal system in Parkinson’s disease. Brain 136, 2419–2431. 10.1093/brain/awt192 23884810 PMC3722357

[B33] Koyano F. Okatsu K. Kosako H. Tamura Y. Go E. Kimura M.. (2014). Ubiquitin is phosphorylated by PINK1 to activate parkin. Nature 510, 162–166. 10.1038/nature13392 24784582

[B34] Laurie D. J. Schoeffter P. Wiederhold K. H. Sommer B. (1997). Cloning, distribution and functional expression of the human mGlu6 metabotropic glutamate receptor. Neuropharmacology 36, 145–152. 10.1016/s0028-3908(96)00172-4 9144651

[B35] Lazarou M. Sliter D. A. Kane L. A. Sarraf S. A. Wang C. Burman J. L.. (2015). The ubiquitin kinase PINK1 recruits autophagy receptors to induce mitophagy. Nature 524, 309–314. 10.1038/nature14893 26266977 PMC5018156

[B36] Lee J. Y. Lee E. K. Park S. S. Lim J. Y. Kim H. J. Kim J. S.. (2009). Association of DRD3 and GRIN2B with impulse control and related behaviors in Parkinson’s disease. Mov. Disord. 24, 1803–1810. 10.1002/mds.22678 19562769

[B37] Liu Q. Wong-Riley M. T. (2010). Postnatal development of N-methyl-D-aspartate receptor subunits 2A, 2B, 2C, 2D and 3B immunoreactivity in brain stem respiratory nuclei of the rat. Neuroscience 171, 637–654. 10.1016/j.neuroscience.2010.09.055 20887777 PMC2987514

[B38] Marcaggi P. Billups D. Attwell D. (2003). The role of glial glutamate transporters in maintaining the independent operation of juvenile mouse cerebellar parallel fibre synapses. J. Physiol. 552, 89–107. 10.1113/jphysiol.2003.044263 12878755 PMC2343331

[B39] Monyer H. Burnashev N. Laurie D. J. Sakmann B. Seeburg P. H. (1994). Developmental and regional expression in the rat brain and functional properties of four NMDA receptors. Neuron 12, 529–540. 10.1016/0896-6273(94)90210-0 7512349

[B40] Narendra D. P. Jin S. M. Tanaka A. Suen D. F. Gautier C. A. Shen J.. (2010). PINK1 is selectively stabilized on impaired mitochondria to activate Parkin. PLoS. Biol. 8:e1000298. 10.1371/journal.pbio.1000298 20126261 PMC2811155

[B41] Ordureau A. Heo J. M. Duda D. M. Paulo J. A. Olszewski J. L. Yanishevski D.. (2015). Defining roles of PARKIN and ubiquitin phosphorylation by PINK1 in mitochondrial quality control using a ubiquitin replacement strategy. Proc. Natl. Acad. Sci. U S A 112, 6637–6642. 10.1073/pnas.1506593112 25969509 PMC4450373

[B42] Park M. Kitahama K. Geffard M. Maeda T. (2000). Postnatal development of the dopaminergic neurons in the rat mesencephalon. Brain Dev. 22, S38–S44. 10.1016/s0387-7604(00)00145-5 10984659

[B43] Pearlstein E. Gouty-Colomer L. A. Michel F. J. Cloarec R. Hammond C. (2015). Glutamatergic synaptic currents of nigral dopaminergic neurons follow a postnatal developmental sequence. Front. Cell. Neurosci. 9:210. 10.3389/fncel.2015.00210 26074777 PMC4448554

[B44] Piccini P. Burn D. J. Ceravolo R. Maraganore D. Brooks D. J. (1999). The role of inheritance in sporadic Parkinson’s disease: evidence from a longitudinal study of dopaminergic function in twins. Ann. Neurol. 45, 577–582. 10.1002/1531-8249(199905)45:5<577::aid-ana5>3.0.co;2-o 10319879

[B45] Piccini P. Morrish P. K. Turjanski N. Sawle G. V. Burn D. J. Weeks R. A.. (1997). Dopaminergic function in familial Parkinson’s disease: a clinical and 18F-dopa positron emission tomography study. Ann. Neurol. 41, 222–229. 10.1002/ana.410410213 9029071

[B46] Poole A. C. Thomas R. E. Andrews L. A. McBride H. M. Whitworth A. J. Pallanck L. J. (2008). The PINK1/Parkin pathway regulates mitochondrial morphology. Proc. Natl. Acad. Sci. U S A 105, 1638–1643. 10.1073/pnas.0709336105 18230723 PMC2234197

[B47] Schmitz Y. Luccarelli J. Kim M. Wang M. Sulzer D. (2009). Glutamate controls growth rate and branching of dopaminergic axons. J. Neurosci. 29, 11973–11981. 10.1523/JNEUROSCI.2927-09.2009 19776283 PMC2818361

[B48] Shen J. (2010). Impaired neurotransmitter release in Alzheimer’s and Parkinson’s diseases. Neurodegener. Dis. 7, 80–83. 10.1159/000285511 20173332 PMC2859234

[B49] Silvestri L. Caputo V. Bellacchio E. Atorino L. Dallapiccola B. Valente E. M.. (2005). Mitochondrial import and enzymatic activity of PINK1 mutants associated to recessive parkinsonism. Hum. Mol. Genet. 14, 3477–3492. 10.1093/hmg/ddi377 16207731

[B50] Smith I. D. Grace A. A. (1992). Role of the subthalamic nucleus in the regulation of nigral dopamine neuron activity. Synapse 12, 287–303. 10.1002/syn.890120406 1465742

[B51] Stuber G. D. Hnasko T. S. Britt J. P. Edwards R. H. Bonci A. (2010). Dopaminergic terminals in the nucleus accumbens but not the dorsal striatum corelease glutamate. J. Neurosci. 30, 8229–8233. 10.1523/JNEUROSCI.1754-10.2010 20554874 PMC2918390

[B52] Suarez F. Zhao Q. Monaghan D. T. Jane D. E. Jones S. Gibb A. J. (2010). Functional heterogeneity of NMDA receptors in rat substantia nigra pars compacta and reticulata neurones. Eur. J. Neurosci. 32, 359–367. 10.1111/j.1460-9568.2010.07298.x 20618827 PMC4177768

[B53] Tecuapetla F. Patel J. C. Xenias H. English D. Tadros I. Shah F.. (2010). Glutamatergic signaling by mesolimbic dopamine neurons in the nucleus accumbens. J. Neurosci. 30, 7105–7110. 10.1523/JNEUROSCI.0265-10.2010 20484653 PMC3842465

[B54] Tepper J. M. Damlama M. Trent F. (1994). Postnatal changes in the distribution and morphology of rat substantia nigra dopaminergic neurons. Neuroscience 60, 469–477. 10.1016/0306-4522(94)90258-5 7915412

[B55] Valente E. M. Salvi S. Ialongo T. Marongiu R. Elia A. E. Caputo V.. (2004). PINK1 mutations are associated with sporadic early-onset parkinsonism. Ann. Neurol. 56, 336–341. 10.1002/ana.20256 15349860

[B56] Wang H. L. Chou A. H. Wu A. S. Chen S. Y. Weng Y. H. Kao Y. C.. (2011). PARK6 PINK1 mutants are defective in maintaining mitochondrial membrane potential and inhibiting ROS formation of substantia nigra dopaminergic neurons. Biochim. Biophys. Acta 1812, 674–684. 10.1016/j.bbadis.2011.03.007 21421046

[B57] Wenzel A. Villa M. Mohler H. Benke D. (1996). Developmental and regional expression of NMDA receptor subtypes containing the NR2D subunit in rat brain. J. Neurochem. 66, 1240–1248. 10.1046/j.1471-4159.1996.66031240.x 8769890

[B58] Yamaguchi T. Sheen W. Morales M. (2007). Glutamatergic neurons are present in the rat ventral tegmental area. Eur. J. Neurosci. 25, 106–118. 10.1111/j.1460-9568.2006.05263.x 17241272 PMC3209508

[B60] Yamaguchi T. Wang H. L. Li X. Ng T. H. Morales M. (2011). Mesocorticolimbic glutamatergic pathway. J. Neurosci. 31, 8476–8490. 10.1523/JNEUROSCI.1598-11.2011 21653852 PMC6623324

[B59] Yamaguchi T. Wang H. L. Morales M. (2013). Glutamate neurons in the substantia nigra compacta and retrorubral field. Eur. J. Neurosci. 38, 3602–3610. 10.1111/ejn.12359 24102658 PMC3903463

[B61] Yang Y. Ouyang Y. Yang L. Beal M. F. McQuibban A. Vogel H.. (2008). Pink1 regulates mitochondrial dynamics through interaction with the fission/fusion machinery. Proc. Natl. Acad. Sci. U S A 105, 7070–7075. 10.1073/pnas.0711845105 18443288 PMC2383971

[B62] Zainal Abidin S. Tan E. L. Chan S. C. Jaafar A. Lee A. X. Abd Hamid M. H.. (2015). DRD and GRIN2B polymorphisms and their association with the development of impulse control behaviour among Malaysian Parkinson’s disease patients. BMC. Neurol. 15:59. 10.1186/s12883-015-0316-2 25896831 PMC4417293

[B63] Zhang S. Qi J. Li X. Wang H. L. Britt J. P. Hoffman A. F.. (2015). Dopaminergic and glutamatergic microdomains in a subset of rodent mesoaccumbens axons. Nat. Neurosci. 18, 386–392. 10.1038/nn.3945 25664911 PMC4340758

[B64] Zhou C. Huang Y. Przedborski S. (2008). Oxidative stress in Parkinson’s disease: a mechanism of pathogenic and therapeutic significance. Ann. N Y Acad. Sci. 1147, 93–104. 10.1196/annals.1427.023 19076434 PMC2745097

